# LncRNA PVT1 is increased in renal cell carcinoma and affects viability and migration in vitro

**DOI:** 10.1002/jcla.24442

**Published:** 2022-04-20

**Authors:** Julia Bohosova, Marek Kasik, Adela Kubickova, Karolina Trachtova, Michal Stanik, Alexandr Poprach, Ondrej Slaby

**Affiliations:** ^1^ 37748 Central European Institute of Technology Masaryk University Brno Czech Republic; ^2^ Department of Urology The University Hospital Brno, Faculty of Medicine, Masaryk University Brno Czech Republic; ^3^ 37748 Department of Pharmacology Faculty of Medicine Masaryk University Brno Czech Republic; ^4^ Department of Urologic Oncology, Department of Comprehensive Cancer Care Faculty of Medicine Masaryk Memorial Cancer Institute Masaryk University Brno Czech Republic; ^5^ 37748 Department of Biology Faculty of Medicine Masaryk University Brno Czech Republic

**Keywords:** diagnosis, long non‐coding RNA, migration next‐generation sequencing, proliferation, transcriptome

## Abstract

**Background:**

Renal cell carcinoma is difficult to diagnose and unpredictable in disease course and severity. There are no specific biomarkers for diagnosis and prognosis estimation feasible in clinical practice. Long non‐coding RNAs (lncRNAs) have emerged as potent regulators of gene expression in recent years. Aside from their cellular role, their expression patterns could be used as a biomarker of ongoing pathology.

**Methods:**

In this work, we used next‐generation sequencing for global lncRNA expression profiling in tumor and non‐tumor tissue of RCC patients. The four candidate lncRNAs have been further validated on an independent cohort. PVT1, as the most promising lncRNA, has also been studied using functional in vitro tests.

**Results:**

Next‐generation sequencing showed significant dysregulation of 1163 lncRNAs; among them top 20 dysregulated lncRNAs were AC061975.7, AC124017.1, AP000696.1, AC148477.4, LINC02437, GATA3‐AS, LINC01762, LINC01230, LINC01271, LINC01187, LINC00472, AC007849.1, LINC00982, LINC01543, AL031710.1, and AC019197.1 as down‐regulated lncRNAs; and SLC16A1‐AS1, PVT1, LINC0887, and LUCAT1 as up‐regulated lncRNAs. We observed statistically significant dysregulation of PVT1, LUCAT1, and LINC00982. Moreover, we studied the effect of artificial PVT1 decrease in renal cell line 786–0 and observed an effect on cell viability and migration.

**Conclusion:**

Our results show not only the diagnostic but also the therapeutic potential of PVT1 in renal cell carcinoma.

## Graphical abstract

We observed statistically significant dysregulation of PVT1, LUCAT1, and LINC00982. Moreover, we studied the effect of artificial PVT1 decrease in renal cell line 786–0 and observed an effect on cell viability and migration. Our results show not only the diagnostic but also the therapeutic potential of lncRNAs.

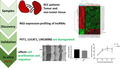

## 1 INTRODUCTION

As the most common malignancy of the kidney, renal cell carcinoma represents more than 2% of all new cancer cases, with almost 200,000 deaths per year worldwide.^1^ Due to the lack of specific symptoms, renal cell carcinoma is often diagnosed at an advanced stage when its treatment becomes more difficult. Usually, the diagnosis happens unexpectedly during the examination indicated for other RCC‐unrelated health problems. At present, there are no reliable biomarkers for diagnosing RCC with sufficient sensitivity and specificity, either at an early or advanced stage or which would help predict the development of the disease without or with therapy.^2^

In recent decades, long non‐coding RNAs (lncRNAs) emerged as potential biomarkers of cancer and other diseases. Specifically in cancer, the role of lncRNAs is currently being addressed by many research groups. LncRNAs act as oncogenes and tumor suppressors and are involved in various signaling pathways.^3^ Deregulation of lncRNA expression was also detected in RCC and correlated with clinicopathological data, including tumor stage, degree of differentiation, or the presence of metastases.^4^, ^5^, ^6^ Many lncRNAs are considered not only diagnostic markers, but their expression is also essential in determining prognosis or monitoring response to treatment.^4^

The present study aimed to investigate the lncRNA expression profile using a next‐generation sequencing approach with subsequent validation of the results. Moreover, we provided some functional in vitro tests of PVT1 as one of the most significantly dysregulated lncRNAs in our patient cohort.

## 2 MATERIAL AND METHODS

### 2.1 Samples and patients

Samples were collected at Masaryk Memorial Cancer Institute, Brno, the Czech Republic, between 2004 and 2014, according to the Declaration of Helsinki with the signed informed consent from all enrolled patients. Ethics Committee of the Masaryk Memorial Cancer Institute and Ethics Committee of the Masaryk University approved the study. The renal parenchyma and paired healthy non‐tumor tissue samples were collected from 46 patients with renal cell carcinoma during nephrectomy. The tissue was snap frozen and stored at −80°C until further processing. Select clinical characteristics of the samples are shown in Table 1.

**TABLE 1:** Select clinical characteristics of the patient cohort

Characteristic	Cohort 1—sequencing	Cohort 2—validation
Number of patients	16	30
Sex		
Women	4	13
Men	12	17
Sample type		
Tumor tissue	16	30
Non‐tumor tissue	6	30
Age at the time of the diagnosis		
Median (years)	63	64
Range (years)	40–81	42–79
Relapse	4	4
Stage		
I	4	14
II	1	7
III	6	7
IV	5	2
Grade		
1	2	4
2	4	13
3	9	10
4	1	3

### 2.2 RNA extraction and quality measurement

Total RNA enriched for small RNAs was extracted using mirVana™ miRNA Isolation kit (Invitrogen) according to the manufacturer's protocol from all collected samples. RNA concentration was measured using Qubit™ 2.0 (Invitrogen, Thermo Fisher Scientific) fluorometer. According to the manufacturer protocol, RNA integrity has been measured in all samples using Agilent 2200 TapeStation system and RNA ScreenTape (Agilent). For the sequencing analysis, 22 samples with the best RNA integrity (RIN >6, average 7.8) from 16 RCC patients (16 tumor tissue and 6 non‐tumor tissue samples) were selected due to the high demands of RNA library preparation for the quality of the material.

### 2.3 Library preparation and RNA sequencing

According to the results from TapeStation, some samples had a significant amount of genomic DNA, which would be problematic during the transcriptomic library preparation. Thus, the genomic DNA had to be removed using DNA‐free™ DNA Removal kit (Invitrogen, Thermo Fisher Scientific) according to the manufacturer's protocol. RNA concentration was again measured using Qubit™ 2.0 (Invitrogen, Thermo Fisher Scientific) fluorometer. After that, RNA was diluted in 26 µl of nuclease‐free water (Qiagen) in the required range 1–1000 ng of total RNA. As the entry amount concentration, 500 ng of RNA was chosen in our case. Using RiboCop rRNA Depletion Kit V1.2 (Lexogen), according to the manufacturer's protocol, ribosomal depletion has been carried out to eliminate ribosomal RNA, which represents the majority of the RNA content and thus would compromise the sequencing capacity after the library preparation. After the ribosomal depletion, the RNA concentration was measured again using Qubit™ 2.0 (Invitrogen, Thermo Fisher Scientific) fluorometer.

Sequencing libraries have been prepared using NEBNext^®^ Ultra™ II Directional RNA Library Prep Kit for Illumina^®^ (New England Biolabs), AM‐Pure^®^ XP Beads (Beckman Coulter), and a combination of index primers NEBNext^®^ Multiplex Oligos for Illumina^®^ (Index Primers Set 1) (New England Biolabs) and NEBNext^®^ Multiplex Oligos for Illumina^®^ (Dual Index Primers Set 1) (New England Biolabs) so they mutually do not collide in different samples. Library preparation has been carried out according to the manufacturer's protocol with minor adjustments: the fragmentation time in the RNA fragmentation and priming step has been set to 8 min (despite higher RNA quality); incubation with the USER enzyme after the adaptor ligation has been skipped and carried out as a first in the PCR enrichment of adaptor ligated DNA reaction; moreover, we decided to run the PCR in the Biometra Optical Thermocycler^®^ (Analytik Jena) which allows following the amplification curve in real‐time and pausing the PCR reaction when individual samples reach the desired signal amount. Therefore, we added 2 µl of EvaGreen^®^ Dye, 20× in Water (Biotium) into the PCR reaction mix. Individual microtubes were taken out of the thermocycler when the amplification curve reached the 5000 fluorescence limit. Purifying beads volume was adjusted because the volume of the product was lower before the PCR and higher after the PCR. Prepared libraries have been stored at −20°C until further processing.

The libraries' quality and quantity have been measured using the Qubit™ 2.0 (Invitrogen, Thermo Fisher Scientific) fluorometer and Agilent 2200 TapeStation system and High Sensitivity D1000 ScreenTape (Agilent) according to the manufacturer's protocol. The libraries have been pooled equimolar at the 4 nM concentration. The concentration was rechecked using the Qubit™ 2.0 (Invitrogen, Thermo Fisher Scientific) fluorometer. The size of the pool was checked using Agilent 2200 TapeStation system and High Sensitivity D1000 ScreenTape (Agilent) according to the manufacturer's protocol. As the libraries contained some fragments of undesirable length (over 650 bp), which were visible at the pool electropherogram, those have been removed using PippinPrep (Sage Science).

According to the Illumina denature and dilute protocol, the pool has been denatured and diluted to 1.8 pM and loaded onto a sequencing cassette from NextSeq 500/550 High Output v2 kit, 75 cycles (Illumina), and run according to the manufacturer's protocol.

### 2.4 Data analysis

Raw data from the Illumina NextSeq 500 were converted to fastq using bcl2fastq2 Con‐version software (version 2.20.0), and read quality was checked using FastQC (version 0.11.7).^7^ Adapter sequences were identified using the Kraken system (version 15‐065),^8^ and poor read ends were removed using Cutadapt (version 1.18).^9^ The 3′ ends with a threshold value less than five and reads shorter than 35 bp have been considered poor and were removed. The modified libraries were mapped with the STAR tool (version 2.7.0d)^10^ to the human genome (GRCh38), the sequence of which was downloaded from the Ensembl database. During mapping, each reading was allowed to map to up to 20 different locations. Genes were quantified using RSEM software (version 1.3.1),^11^ and differentially expressed lncRNAs were identified using the DESeq2 tool (version 1.18.1).^12^ LncRNAs with a fold change higher than 2 and an adjusted p‐value less than 0.05 were considered differentially expressed. For the validation, 4 lncRNAs within the 20 most dysregulated lncRNAs have been chosen, considering the previously published results on individual candidates.

### 2.5 Validation and statistical analysis

Validation of the results from the exploratory phase has been carried out using a High‐Capacity cDNA Reverse Transcription Kit (Applied Biosystems, Thermo Fisher Scientific) for reverse transcription. TaqMan™ Gene Expression Master Mix (Applied Biosystems, Thermo Fisher Scientific) was used in qPCR run on QuantStudio 12K Flex Real‐Time PCR System (Applied Biosystems, Thermo Fisher Scientific) according to the manufacturer's protocol. Following TaqMan^®^ Gene Expression Assays were used for qPCR: PVT1, LUCAT1, LINC00982, and PPIA (Applied Biosystems, Thermo Fisher Scientific). Cq values have been normalized to the expression level of PPIA as a reference gene based on the literature search and our preliminary measurements of PPIA stability in RCC tissue specimens.

Normalized expression data were statistically evaluated using Mann–Whitney U Test, Wilcoxon test, Kruskal–Wallis test, ROC analysis, and Kaplan–Meier analysis (GraphPad Prism 5, GraphPad Software).

### 2.6 Cell culture and transfection

The human renal cell carcinoma cell line 786‐0 (ATCC^®^ CRL‐1932™), renal adenocarcinoma cell line Achn (ATCC^®^ CRL‐1611™), and clear cell renal cell carcinoma cell line Caki‐2 (ATCC^®^ HTB‐47™) were obtained from the American Type Culture Collection. The 786–0 cells were cultured in Rosewell‐Park Memorial Institute (RPMI) medium with 10% FBS, 100 μg/ml penicillin, and 100 μg/ml streptomycin (Invitrogen, Gibco) in 5% CO2 at 37°C. The Achn cells were cultured in Eagle's Minimum Essential Medium with 10% FBS, 100 μg/ml penicillin, and 100 μg/ml streptomycin (Invitrogen, Gibco) in 5% CO_2_ at 37°C. The Caki‐2 cells were cultures in McCoy's 5a Medium Modified with the addition of 10% FBS, 100 μg/ml penicillin, and 100 μg/ml streptomycin (Invitrogen, Gibco) in 5% CO_2_ at 37°C.

Cells were plated in a 24‐well plate at a density of 25 × 10^3^ cells per well 24 h before transfection. Subsequently, the cells were transfected with 10 pM of Silencer™ Select Pre‐Designed siRNA (ID: n272515, n272517, and n272521; Ambion, Thermo Fisher Scientific) or Silencer™ Select Negative Control No. 1 siRNA (Invitrogen, Thermo Fisher Scientific) and an equimolar amount of Lipofectamine™ RNAiMAX Reagent (Invitrogen, Thermo Fisher Scientific).

### 2.7 RNA extraction, reverse transcription, and real‐time PCR

Transfected cells were harvested 24, 48, 72, and 96 h after transfection. Total RNA was extracted using Direct‐zol RNA MiniPrep (Zymo Research) isolation kit according to the manufacturer's protocol. The concentration and purity of extracted RNA were determined using Nanodrop 2000c (Thermo Scientific). Reverse transcription and real‐time PCR were performed using High‐Capacity cDNA Reverse Transcription Kit (Applied Biosystems, Thermo Fisher Scientific) for reverse transcription and TaqMan™ Gene Expression Master Mix (Applied Biosystems, Thermo Fisher Scientific) and TaqMan^®^ Gene Expression Assay (PVT1) (Applied Biosystems, Thermo Fisher Scientific) for qPCR on QuantStudio 12K Flex Real‐Time PCR System (Applied Biosystems, Thermo Fisher Scientific) according to the manufacturer's protocol. Cq values have been normalized to the expression level of PPIA as a reference gene. All data were analyzed using the 2^−ΔCt^ method.

### 2.8 Cell proliferation assay

Cell proliferation was determined using the cell counting method. The 786–0 cells were seeded in a 24‐well plate at the density of 25 × 10^3^ cells per well 24 h before transfection. Subsequently, the cells were transfected with 10 pM of Silencer™ Select Pre‐Designed siRNA (ID: n272515; Ambion, Thermo Fisher Scientific) or Silencer™ Select Negative Control No. 1 siRNA (Invitrogen, Thermo Fisher Scientific) and an equimolar amount of LipofectamineTM RNAiMAX Reagent (Invitrogen, Thermo Fisher Scientific). Cells were harvested and counted in the Bürker chamber after 24, 48, 72, and 96 h after transfection.

### 2.9 Scratch wound migration assay

The 786–0 cells were plated in a 24‐well plate at the density of 25 × 10^4^ cells per well 24 h before transfection with 10 pM Silencer™ PVT1 siRNA or Silencer™ Select Negative Control No. 1 siRNA. After 24 h since transfection, 50 µl of mitomycin was added to each well, and the plate was incubated for 1 h at 37°C with 5% CO_2_. Subsequently, the cell monolayer was disturbed using a sterile pipette tip and rinsed with PBS removing cell debris. PBS was removed and replaced with the fresh medium. The migration was measured right after wounding and 12 h later with Nikon Diaphod 300 INV (×10) and camera Canon Power shot A95. Images were analyzed by the Tscratch software (CSE).

## 3 RESULTS

### 3.1 Expression profiling in the tissue of RCC patients

**FIGURE 1:**
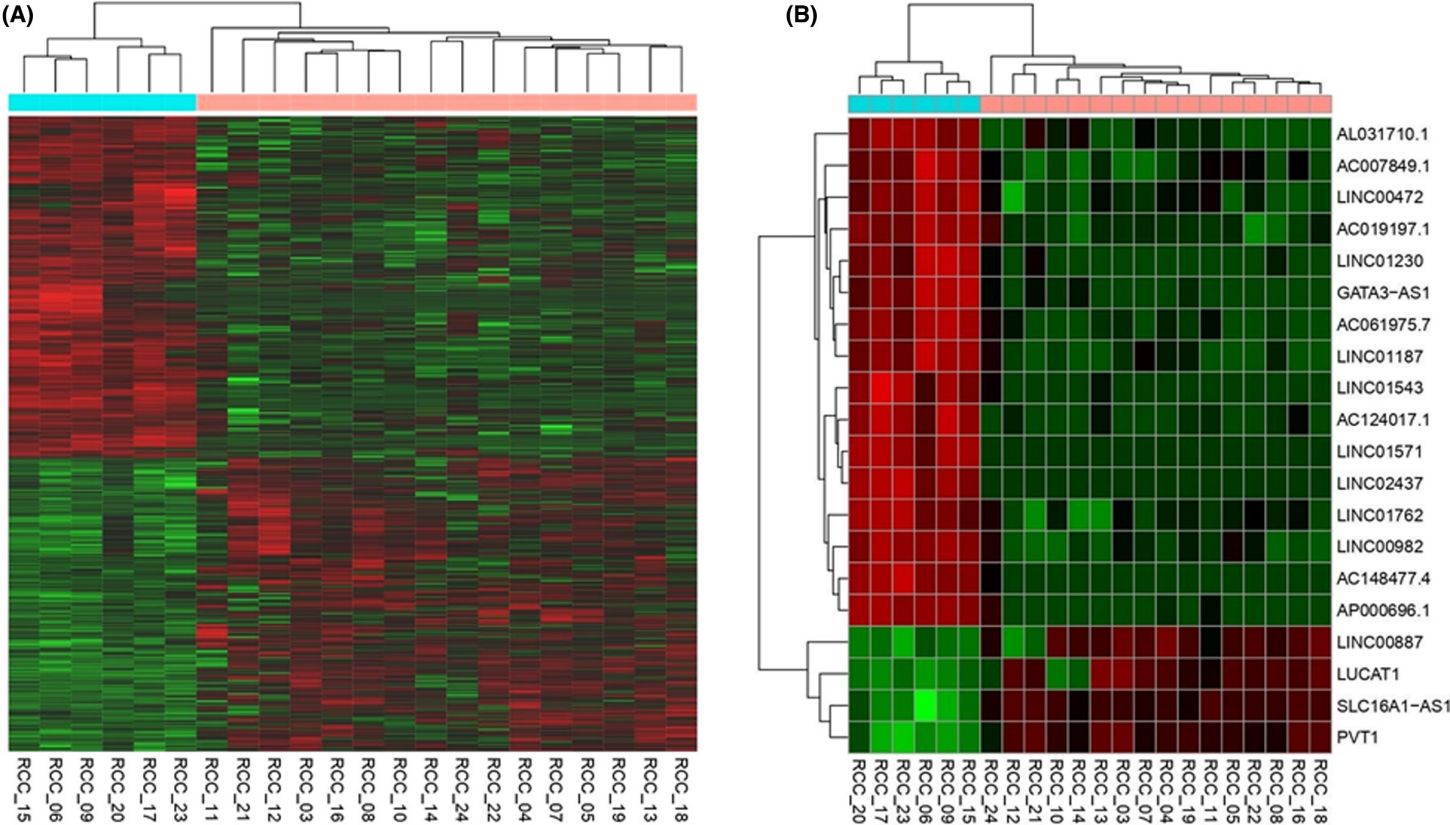
Clastrogram and the heatmap showing (A) 1163 significantly dysregulated lncRNAs and (B) showing 20 most significantly dysregulated lncRNAs in 16 samples of tumor tissue (pink) and 6 non‐tumor tissue samples (blue). In the heatmap, red shows higher expression, and green shows lower expression. LncRNAs with fold change >2 and *p* < 0.05 were considered significant

Using next‐generation sequencing and the DESeq2 tool, we analyzed lncRNA expression profile in tumor and non‐tumor renal parenchyma. We identified 1163 dysregulated lncRNAs in renal tumor tissue, 538 up‐regulated and 625 down‐regulated (Figure 1A). The 20 most significantly dysregulated lncRNAs are shown in the heatmap (Figure 1B) and listed in Table 2.

**TABLE 2:** Twenty most significantly dysregulated lncRNAs in tumor and non‐tumor renal tissue of RCC patients according to the *p*‐value and adjusted *p*‐value; FC—Fold change in relation to the non‐tumor tissue, ↑—up‐regulated in tumor tissue, and ↓—down‐regulated in tumor tissue

Gene ID	Gene name	FC	↑↓	*p*‐Value	Adjusted *p*	Gene biotype
ENSG00000226419	SLC16A1‐AS1	−2.94	↑	5.12283E−31	2.42607E−27	Antisense RNA
ENSG00000267259	AC061975.7	6.65	↓	7.04435E−31	2.42607E−27	lincRNA
ENSG00000249341	AC124017.1	7.89	↓	1.20099E−30	2.75747E−27	Sense intronic
ENSG00000231324	AP000696.1	6.33	↓	1.59356E−29	2.7441E−26	lincRNA
ENSG00000249859	PVT1	−4.31	↑	6.02861E−28	8.30501E−25	lincRNA
ENSG00000277011	AC148477.4	7.01	↓	1.01868E−27	1.16945E−24	lincRNA
ENSG00000248517	LINC02437	7.34	↓	4.34521E−26	4.27568E−23	lincRNA
ENSG00000197308	GATA3‐AS1	7.27	↓	2.10633E−25	1.81355E−22	lincRNA
ENSG00000233154	LINC01762	4.60	↓	3.21509E−25	2.46062E−22	lincRNA
ENSG00000281769	LINC01230	7.85	↓	8.14587E−24	5.61087E−21	lincRNA
ENSG00000260057	LINC01571	6.71	↓	2.40728E−23	1.50739E−20	lincRNA
ENSG00000249601	LINC01187	7.23	↓	1.60518E−21	9.21373E−19	lincRNA
ENSG00000214145	LINC00887	−6.39	↑	1.73399E−20	9.18747E−18	lincRNA
ENSG00000248323	LUCAT1	−6.49	↑	6.90083E−20	3.39521E−17	lincRNA
ENSG00000233237	LINC00472	2.27	↓	7.70808E−19	3.53955E−16	lincRNA
ENSG00000242795	AC007849.1	4.63	↓	1.62448E−18	6.99337E−16	Sense intronic
ENSG00000263862	LINC01543	6.72	↓	4.64644E−18	1.88263E−15	lincRNA
ENSG00000177133	LINC00982	7.42	↓	1.11233E−17	4.25653E−15	Antisense RNA
ENSG00000261399	AL031710.1	4.91	↓	1.48083E−17	5.36841E−15	Antisense RNA
ENSG00000236283	AC019197.1	5.64	↓	1.88796E−17	6.50214E−15	Antisense RNA

**FIGURE 2:**
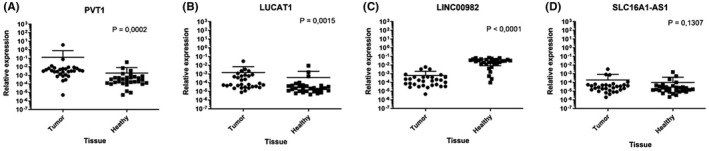
Graph showing expression of candidate lncRNAs in tumor tissue compared to non‐tumor tissue of RCC patients, analyzed using non‐parametric paired Wilcoxon test. (A) Up‐regulation of PVT1, *p* = 0.0002; (B) up‐regulation of LUCAT1, *p* = 0.0015; (C) down‐regulation of LINC00982, *p* < 0.0001; and (D) expression of SLC16A1‐AS1 without significant difference, *p* = 0.1774

Four lncRNA candidates have been selected for the validation—PVT1, LUCAT1, LINC00982, and SLC16A1‐AS1. Their expression has been measured in the paired tumor, and non‐tumor tissue specimens and Cq values were normalized to the expression of PPIA. Normalized values were analyzed statistically using the Wilcoxon test. Results are shown in Figure 2. Significant dysregulation has been observed in the expression of PVT1 (*p* = 0.0002 [Figure 2A]) and LUCAT1 (*p* = 0.0015 [Figure 2B]), which were down‐regulated, and in the expression of LINC00982 (*p* < 0.0001, Figure 2C). No significant difference in expression has been observed in SLC16A1‐AS1 (*p* = 0.1307, Figure 2D).

**FIGURE 3:**
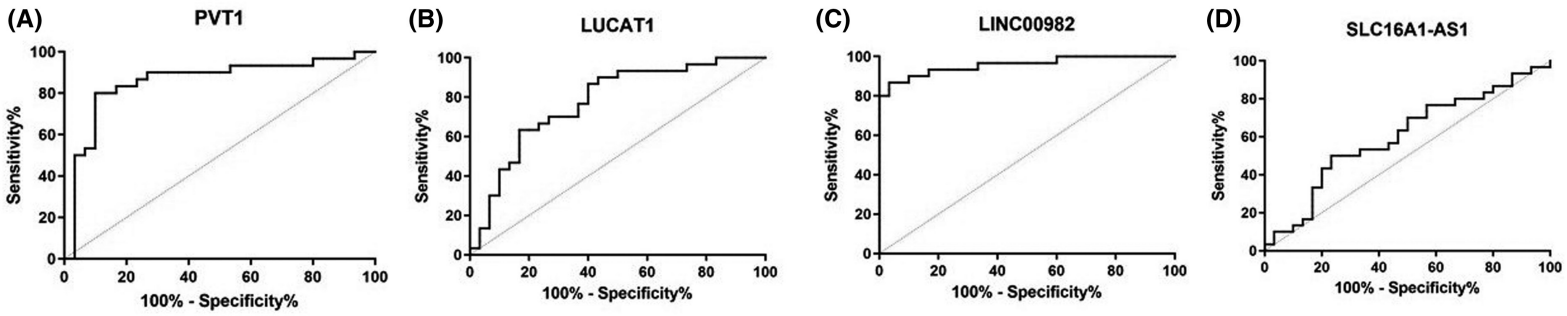
Graph showing ROC curves of candidate lncRNAs in tumor tissue compared to non‐tumor tissue of RCC. (A) ROC curve of PVT1, sensitivity 86.67%, specificity 76.67%, AUC = 0.8567, *p* < 0.0001; (B) ROC curve of LUCAT1, sensitivity 90%, specificity 90%, AUC = 0.7756, *p* = 0.0002; (C) ROC curve of LINC00982, sensitivity 76.67%, specificity 66.67%, AUC = 0.9578, *p* < 0.0001; (D) ROC curve of SLC16A1‐AS1, sensitivity 66.67%, specificity 53.33%, AUC = 0.6022, *p* = 0.1738

In four validated lncRNAs, we also did ROC analysis (Figure 3A–D). All lncRNAs except SLC16A1‐AS1 showed an ability to distinguish tumor and non‐tumor tissue with high sensitivity and specificity and AUC higher than 0.75.

We compared the expression of our four candidate lncRNAs in patients' stage I and in patients with stages II and IV and patients with lower grades (1 and 2) and with higher grades (3 and 4) and found no correlation either for stage or grade (data not shown).

### 3.2 Functional characterization of PVT1 in vitro

**FIGURE 4:**
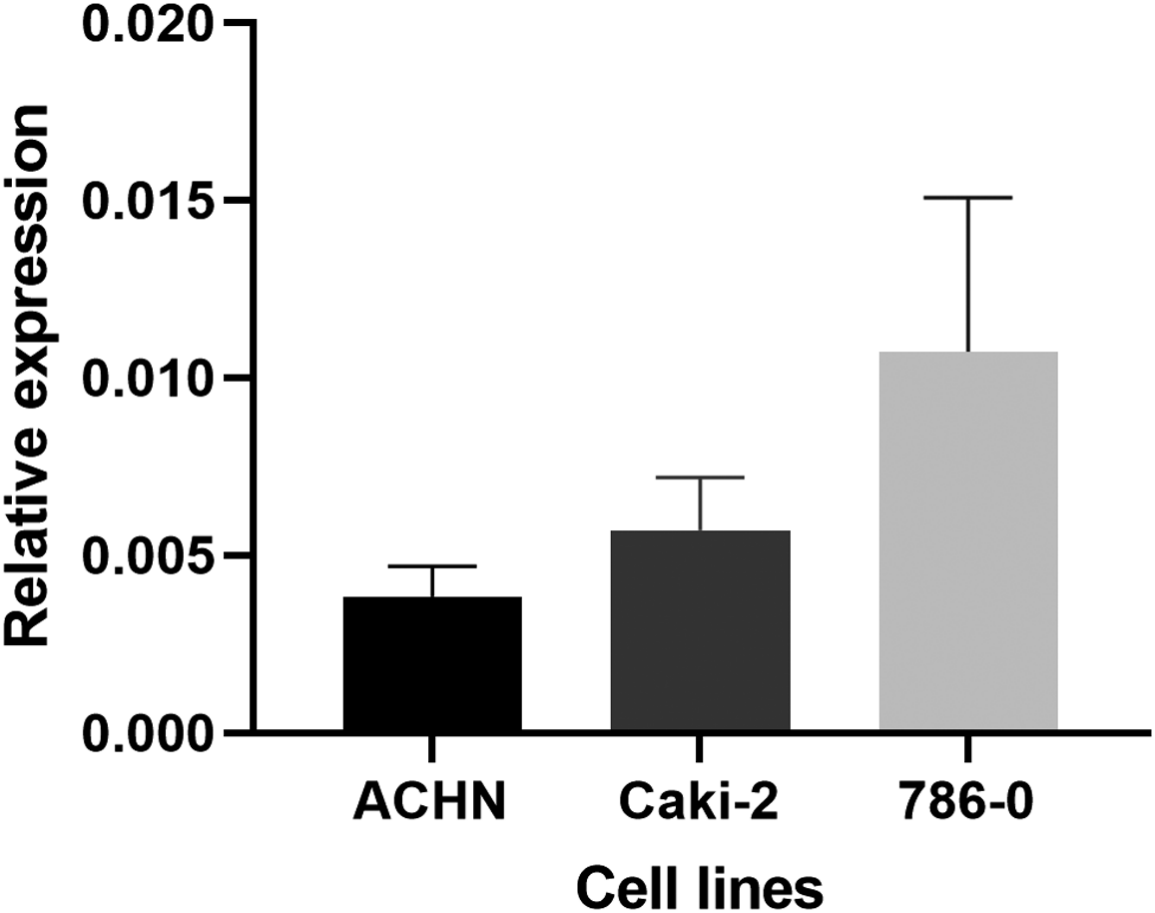
Expression of PVT1 in three selected cell lines

Using qPCR, we measured the expression of PVT1 in three renal cell carcinoma cell lines (ACHN, Caki‐2, and 786–0). PPIA was used as the endogenous control. The highest expression of PVT1 was detected in 786–0 cells. Thus, we chose them as our study model for artificial down‐regulation of PVT1. Results are shown in Figure 4.

**FIGURE 5:**
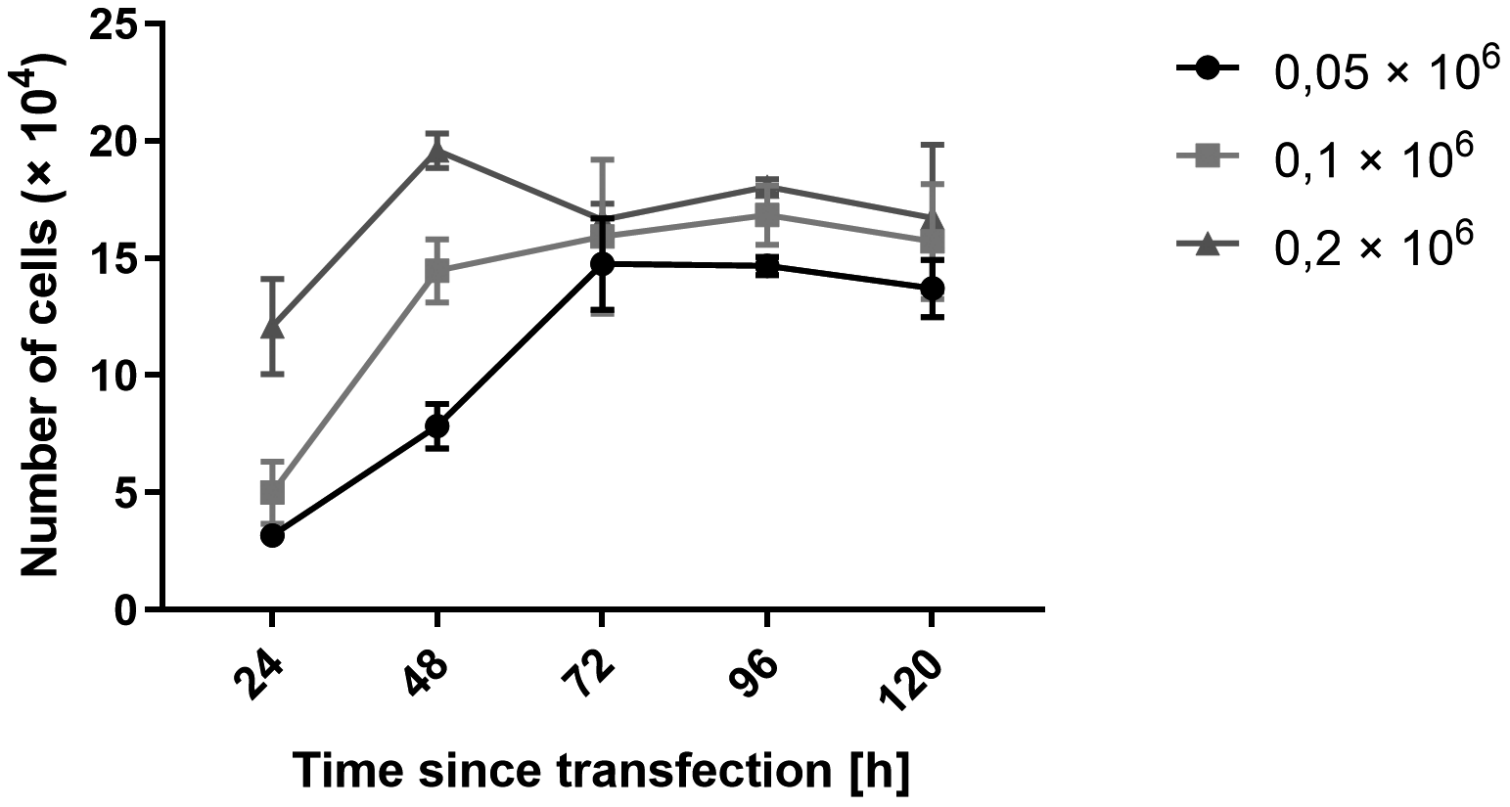
Growth curve of the cell line 786–0. The graph shows average values and standard deviation of the triplicate at each time point and concentration

Furthermore, we created a growth curve of the cell line to determine the appropriate concentration of cells for in vitro experiments. Based on the results, we continued with the concentration 0.1 × 10^6^ cells as the cells reached the logarithmic growth phase within 24–96 h after seeding. Cells in the highest concentration behaved unusually, decreasing after 48 h and increasing after 72 h after seeding. Results are shown in Figure 5.

**FIGURE 6:**
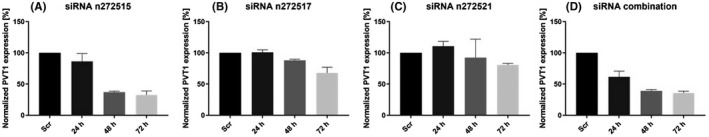
Decrease in PVT1 expression after transfection with different siRNAs. Data are the average values of the triplicates in every time point

Effectivity of transfection has been measured using qPCR 24, 48, and 72 h after transfection using four synthetic oligos (n272515, n272517, n272521, and siRNA mixture) and normalized to expression levels of PPIA. Results are shown in Figure 6A–D. Effect of transfection on levels of PVT1 has been observed only in siRNA n272517 (Figure 6B) immediately after transfection (24 h), as the level of PVT1 decreased to 86.4%. A significant decrease has been observed after 48 h (37.1%) and after 72 h (32.4%), which was more than in the other two siRNAs, where the decrease did not reach 50% at any time point. The siRNA combination also significantly decreased PVT1 (Figure 6D), but this could be mainly ascribed to the effect of siRNA n272517. Therefore, we further continued using this siRNA alone for other experiments.

**FIGURE 7:**
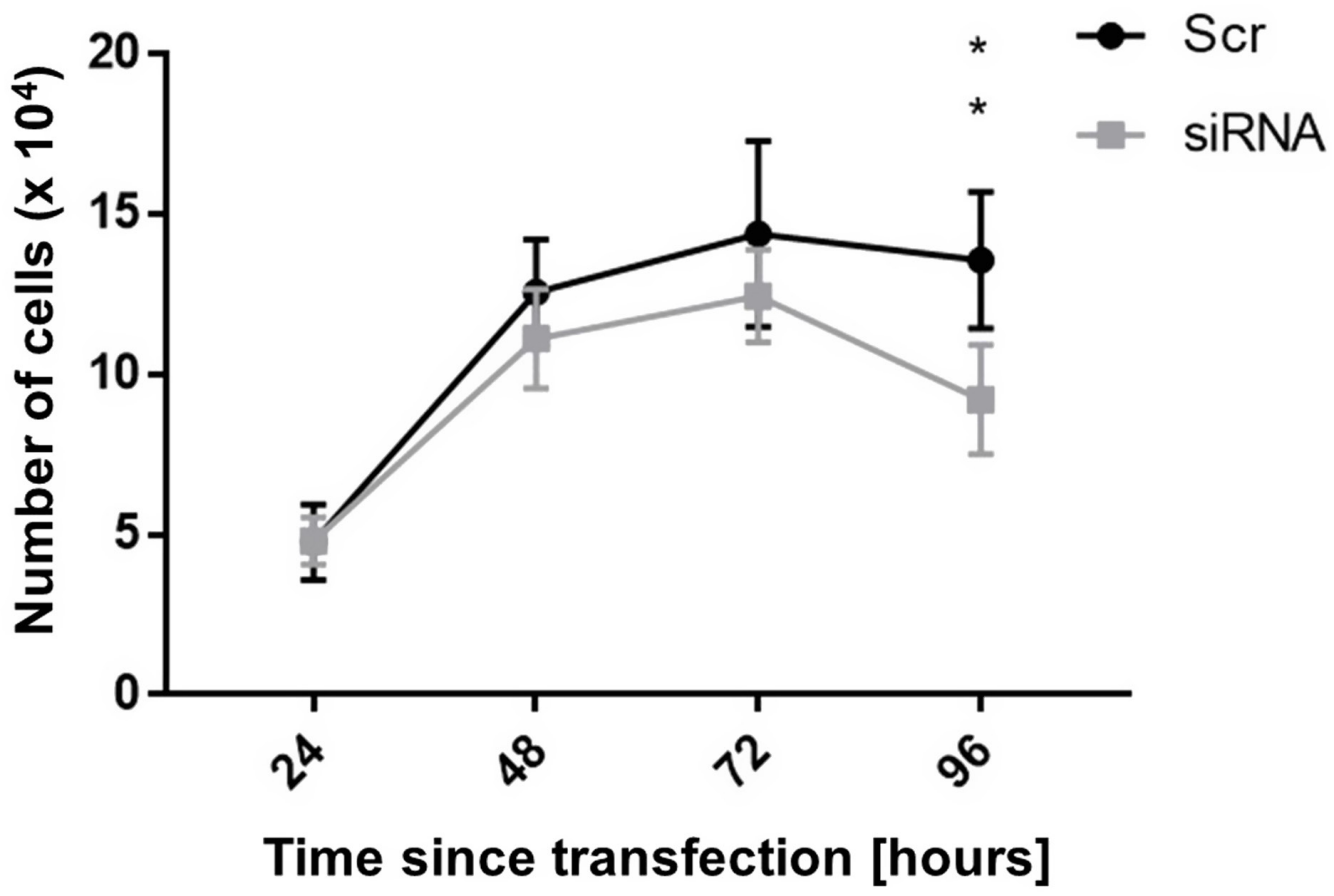
Proliferation profile after transfection with siRNA and control oligonucleotide. Significant PVT1 decrease was observed only 4 days after transfection (**p = 0.0022). The data in the graph are the average values of triplicate in every time point

We used the cell counting technique to assess the effect of PVT1 decrease on proliferation. The cells transfected with control scrambled oligo proliferated more than those transfected with anti‐PVT1 siRNA; however, the statistically significant results have been observed only on the 4th day after transfection (*p* = 0.0022) (Figure 7).

**FIGURE 8:**
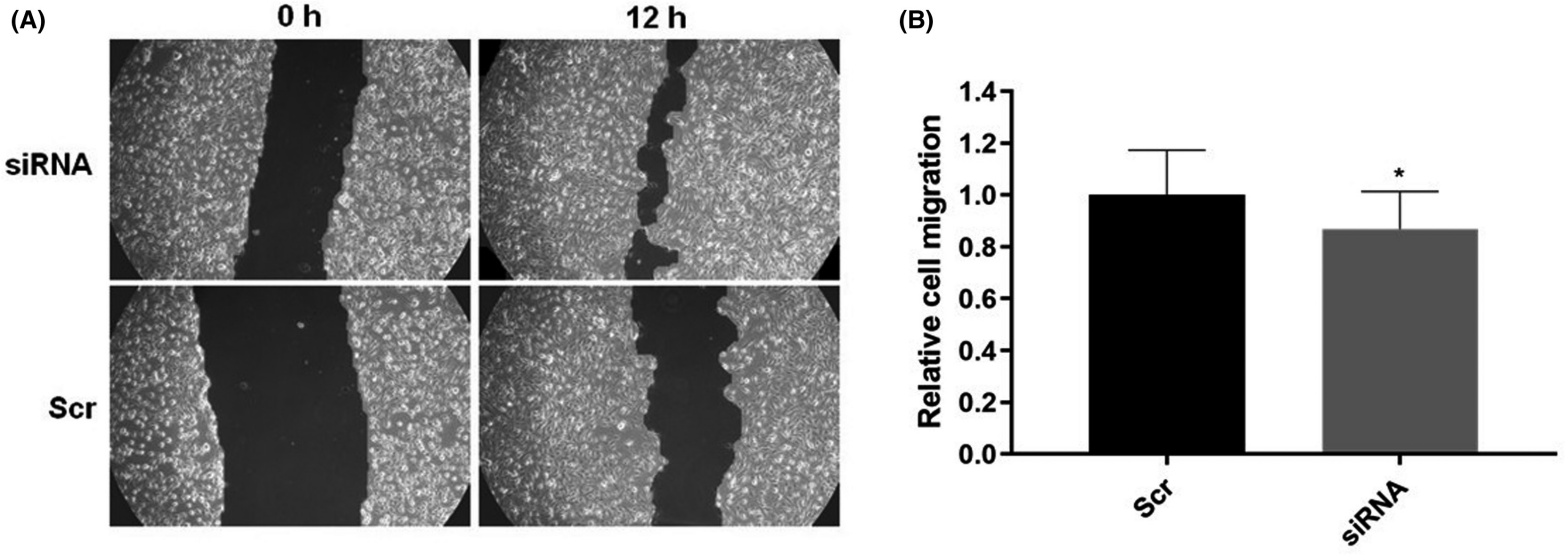
(A) Cell migration was observed using the scratch‐wound assay in T0 and T + 12 h after the wounding. (B) Mann–Whitney *U* test graph showing the results of six analyzed spots in two replicates (*p = 0.0332)

Migration ability has been assessed using the scratch‐wound assay. We observed the regrowth of the cells in the wound created by the pipette tip in T0 and T + 12 h (Figure 8A). Cells transfected with siRNA migrated less than cells transfected with control oligo. Statistical significance has been tested using the Mann–Whitney *U* test (*p* = 0.0332, Figure 8B).

## 4 DISCUSSION

In the present study, our primary goal was to determine the expression profiles of lncRNAs in RCC using next‐generation sequencing (NGS) and validate the expression of selected lncRNAs by qPCR, including determining the association of specific expression profile of these lncRNAs with selected clinicopathological characteristics. The expression of PVT1 and LUCAT1 was significantly increased, and the expression of LINC00982 was significantly decreased in tumor patients, which is in line with available publications analyzing the significance of these lncRNAs not only in RCC but also in other cancers. However, a significant difference between SLC16A1‐AS1 expression in tumor and non‐tumor tissue was not confirmed in the validation phase. The effect of any of the selected lncRNAs on clinicopathological characteristics such as tumor stage and grade could not be identified. Possibly, an effect would be observed on the larger patient cohort.

The most extensively studied lncRNA of our three successfully confirmed candidates is PVT1. Elevated levels of this lncRNA have been detected and described in breast and ovarian cancer, gastric cancer,^13^, ^14^ bladder,^15^ cervix,^16^ esophagus,^17^ non‐small cell lung cancer,^18^, ^19^ or colorectal cancer.^20^ In RCC, PVT1 has an oncogenic effect, and its expression levels are typically increased in the tumor, as shown by several independent works,^18^, ^21^, ^22^, ^23^, ^24^ also reviewed in Bohosova et al.^25^ However, none of these works contains the ROC analysis that would determine the discriminant value of the test based on PVT1 expression. Following the previously published results, PVT1 was also increased in our RCC patient cohort, both in the NGS‐based exploratory phase and in the validation phase using qPCR. We also provided the ROC analysis of the PVT1 diagnostic potential, indicating that this lncRNA is a sufficiently discriminating biomarker capable of distinguishing between tumor and non‐tumor tissue.

On the other hand, the effect of lncRNA on tumor stage and grade was not confirmed, in contrast to previously published studies where a relationship was observed between increased PVT1 expression and tumor stage^18^, ^22^, ^23^, ^24^ and the degree of differentiation.^18^, ^22^, ^23^ This discrepancy between our results and other published studies could be attributed to the small size of our cohort. We could compare only 21 patients with the localized stage (I + II) and 8 patients with the advanced stage (III + IV). Similarly, there were only 17 patients with the lower rate of differentiation (1 + 2) and 12 with a higher rate (3 + 4). A larger cohort would possibly bring significant results similar to the results of our colleagues in other research groups.

Our results were similar in the case of LUCAT1. Increased expression of this lncRNA has been observed in tumor tissue compared to non‐tumor tissue, not only in RCC^26^, ^27^, ^28^, ^29^ but also in other tumors such as non‐small cell carcinoma, lung,^30^ glioma,^31^ clear cell esophageal carcinoma,^32^ or hepatocellular carcinoma.^33^ Following the previous works, we observed significantly increased expression of LUCAT1. No correlation with clinicopathological characteristics has been found. This result contrasts previous results,^26^ where a relationship was observed with both the stage and the degree of differentiation, or only at the stage and other characteristics.^27^, ^28^ The explanation is likely to be similar to PVT1.

The role of LINC00982 was first mentioned in a study by Fei et al.,^34^ where the chip identified reduced levels of this lncRNA in gastric cancer. Its relationship with clinicopathological characteristics and influence on the prognosis of the disease was also described. LINC00982 also has reduced expression levels in lung adenocarcinoma compared to non‐tumor tissue^35^ and also in RCC,^36^ which agrees with our results, indicating that LINC00982 could be a potential diagnostic biomarker of RCC. However, in contrast to Zhang et al.,^36^ where the relationship between decreased LINC00982 expression and tumor stage was described, we did not observe this effect or any relationship to the degree of tumor differentiation.

The second goal was to determine the effect of selected lncRNA using in vitro functional studies. PVT1 lncRNA was chosen because its expression levels indicated oncogenic activity, and the difference in expression between tumor and non‐tumor tissue was the most significant. The 786–0 cell line was selected, and to monitor the effect of PVT1 on cell proliferation and migration, expression of PVT1 was attenuated by transient siRNA transfection. A significant decrease in expression, i.e., more than 50% compared to cells transfected with the control oligonucleotide, was achieved with siRNA n272515, where PVT1 levels were around 37% 48 h after transfection and about 32% after 72 h.

The effect of PVT1 on cell viability was described by Wu et al.,^22^ who studied the 786–0 and ACHN cells' proliferation after suppressing PVT1 expression by transient siRNA transfection. The CCK‐8 assay (Dojindo) was used to test metabolic activity, i.e., cell viability. Transfected cells proliferated less, and their ability to form colonies was reduced. Similar results were obtained in a study by Yang et al.^37^ A lentiviral system suppressed PVT1 expression in the same cell lines, and cell proliferation assays were performed, namely the MTS assay and again the colony formation assay. Li et al.^24^ transfected the 786–0 and Caki‐1 cell lines to reduce PVT1 expression and observed reduced cell proliferation by the MTS assay. Our work confirmed these findings. Cells transfected with siRNA proliferated less than those transfected with the control oligonucleotide, as observed in cell counts in the Bürker chamber. A significant difference was observed only 96 h after transfection, but other time points showed a clear trend. The assay possibly did not suppress PVT1 expression sufficiently to show a clear difference at other time points. The test was performed in triplicate in two independent experiments, although to verify the proliferative capacity of the cells, it would be appropriate to repeat the test several times.

An essential property of tumor cells is their ability to establish metastases. This ability requires cell migration to be tested using a scratch‐wound test or a transwell assay. Yang et al.^37^ observed reduced migratory abilities in cells with experimentally reduced PVT1 expression. By his results, a reduced migration was observed in our work in siRNA‐transfected cells compared to the control oligonucleotide. However, it must be said that the test was performed only in one experiment in duplicate, and to confirm this result, it would be appropriate to repeat the test several times.

Nevertheless, our results contribute to the current knowledge of long non‐coding RNAs in renal cell carcinoma. The distinct expression profiles of lncRNAs in RCC patients were identified, and the expression of selected three of the four lncRNAs was confirmed in an independent group of patients. PVT1, LUCAT1, and LINC00982 thus represent potential diagnostic biomarkers capable of distinguishing between tumor and non‐tumor tissue with high sensitivity and specificity. The importance of PVT1 as a therapeutic target was partially confirmed as we observed that this lncRNA could be involved in the regulation of cell proliferation and migration.

## CONFLICT OF INTEREST

The authors declare no conflict of interest.

## AUTHOR CONTRIBUTIONS

Conceptualization, OS and MS; methodology, OS; validation, JB and AK; formal analysis, MK and JB; resources, OS; data curation, MS and AP; writing—original draft preparation, AK and MK; writing—review and editing, JB; visualization, KT and AK; supervision, OS; and funding acquisition, OS and AP. All authors have read and agreed to the published version of the manuscript.

## Data Availability

Raw sequencing data were generated at CEITEC Genomics Core Facility and are publicly available at the Sequence Read Archive under accession number PRJNA824994. Derived data supporting the findings of this study are available from the corresponding author on request.
